# Deep Learning Techniques for Medical Image Segmentation: Achievements and Challenges

**DOI:** 10.1007/s10278-019-00227-x

**Published:** 2019-05-29

**Authors:** Mohammad Hesam Hesamian, Wenjing Jia, Xiangjian He, Paul Kennedy

**Affiliations:** 1 School of Electrical and Data Engineering (SEDE), University of Technology Sydney, 2007 Sydney, Australia; 2 CB11.09, University of Technology Sydney, 81 Broadway, Ultimo NSW, 2007 Sydney, Australia; 3 School of Software, University of Technology Sydney, 2007 Sydney, Australia

**Keywords:** Deep learning, Medical image segmentation, CNN, Organ segmentation

## Abstract

Deep learning-based image segmentation is by now firmly established as a robust tool in image segmentation. It has been widely used to separate homogeneous areas as the first and critical component of diagnosis and treatment pipeline. In this article, we present a critical appraisal of popular methods that have employed deep-learning techniques for medical image segmentation. Moreover, we summarize the most common challenges incurred and suggest possible solutions.

## Introduction

Medical image segmentation, identifying the pixels of organs or lesions from background medical images such as CT or MRI images, is one of the most challenging tasks in medical image analysis that is to deliver critical information about the shapes and volumes of these organs. Many researchers have proposed various automated segmentation systems by applying available technologies. Earlier systems were built on traditional methods such as edge detection filters and mathematical methods. Then, machine learning approaches extracting hand-crafted features have became a dominant technique for a long period. Designing and extracting these features has always been the primary concern for developing such a system and the complexities of these approaches have been considered as a significant limitation for them to be deployed. In the 2000s, owing to hardware improvement, deep learning approaches came into the picture and started to demonstrate their considerable capabilities in image processing tasks. The promising ability of deep learning approaches has put them as a primary option for image segmentation, and in particular for medical image segmentation. Especially in the previous few years, image segmentation based on deep learning techniques has received vast attention and it highlights the necessity of having a comprehensive review of it. To the best of our knowledge, there is no comprehensive review specifically done on medical image segmentation using deep learning techniques. There are a few recent survey articles on medical image segmentation, such as [49] and [67]. Shen et al. in [67] reviewed various kinds of medical image analysis but put little focus on technical aspects of the medical image segmentation. In [49], many other sections of medical image analysis like classification, detection, and registration is also covered which makes it medical image analysis review not a specific medical image segmentation survey. Due to the vast covered area in this article, the details of networks, capabilities, and shortcomings are missing.

This has motivated us to prepare this article to have an overview of the state-of-art methods. This survey is focusing more on machine learning techniques applied in the recent research on medical image segmentation, has a more in-depth look into their structures and methods and analyzes their strengths and weaknesses.

This article consists of three main sections, approaches (network structures), training techniques, and challenges. The Network Structure section introduces the major, popular network structures used for image segmentation; their advantages; and shortcomings. It is designed to cover the emerging sequence of the structures. Here, we try to address the most significant structures with a major superiority over ancestors. The Training Techniques section explores the state-of-the-art techniques used for training deep neural network models. The Challenges section addresses various types of challenges correlated with medical image segmentation using deep learning techniques. These challenges are mainly related to the design of a network, data, and training. This section also suggests possible solutions according to literature to tackle each of the challenges related to the design of network, data, and training.

## Approaches/Network Structures

### Convolutional Neural Networks (CNNs)

**Fig. 1:**
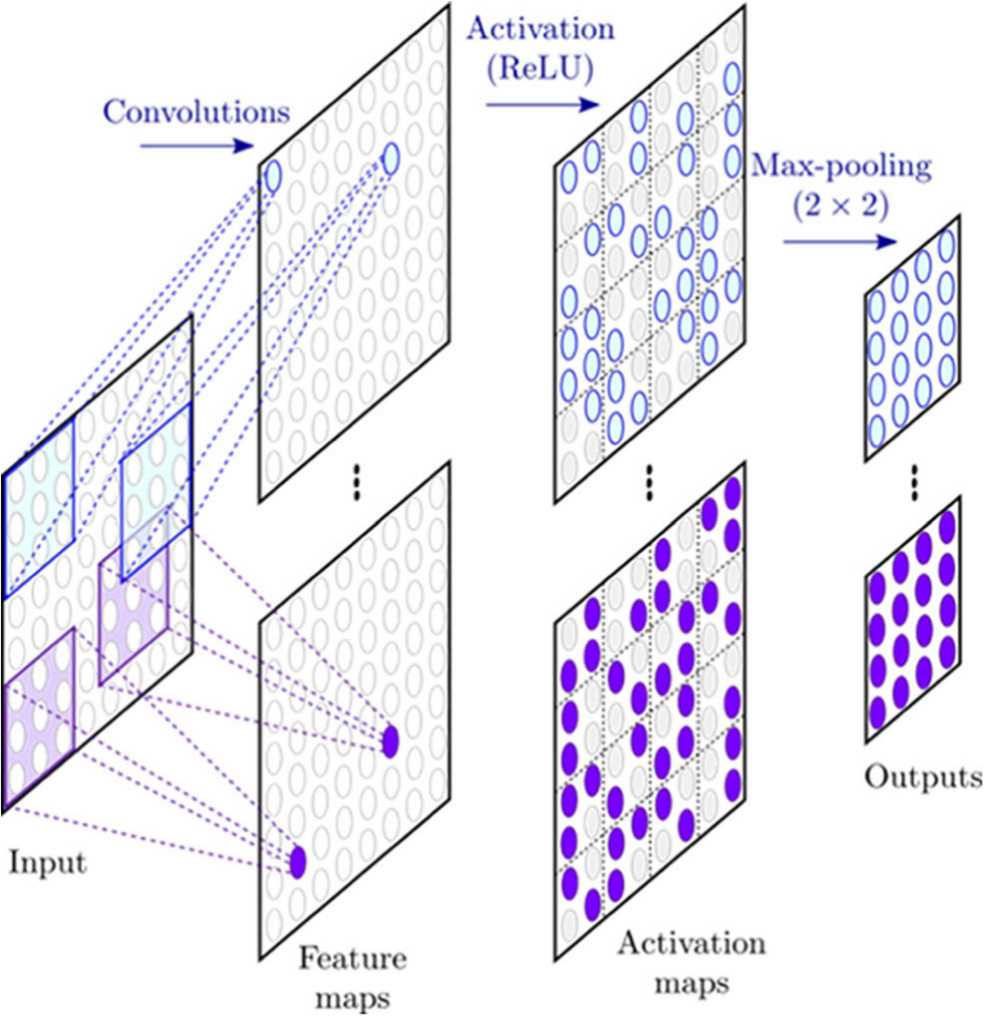
The structure of a CNN [20]

A CNN is a branch of neural networks and consists of a stack of layers each performing a specific operation, e.g., convolution, pooling, loss calculation, etc. Each intermediate layer receives the output of the previous layer as its input (see Fig. 1). The beginning layer is an input layer, which is directly connected to an input image with the number of neurons equal to the number of pixels in the input image. The next set of layers are convolutional layers that present the results of convolving a certain number of filters with the input data and perform as a feature extractor. The filters, commonly known as kernels, are of arbitrary sizes, defined by designers, and depending on the kernel size. Each neuron responds only to a specific area of the previous layer, called receptive field. The output of each convolution layer is considered as an activation map, which highlights the effect of applying a specific filter on the input. Convolutional layers are usually followed by activation layers to apply non-linearity to the activation maps. The next layer can be a pooling layer depending on the design and it helps to reduce the dimensionality of the convolution’s output. To perform the pooling, there are a few strategies, such as max pooling and average pooling. Lastly, high-level abstractions are extracted by fully connected layers. The weights of neural connections and the kernels are continuously optimized during the procedure of a back propagation in the training phase [20].

The above structure is known as a conventional CNN. In the following sub-sections, we review the application of these structures in medical image segmentation.

#### 2D CNN

With the promising capability of a CNN in performing image classification and pattern recognition, applying a CNN to medical image segmentation has been explored by many researchers.

The general idea is to perform segmentation by using a 2D input image and applying 2D filters on it. In the study done by Zhang et al. [89], multiple sources of information (T1, T2, and FA) in the form of 2D images are passed to the input layer of a CNN in various image channels (e.g., R, G, B) to investigate if the use of multi-modality images as input improves the segmentation outcomes. Their results have demonstrated better performance than those using a single modality input. In another experiment done by Bar et al [4], a transfer learning approach is taken into account and low-level features are borrowed from a pre-trained model on Imagenet. The high-level features are taken from PiCoDes [6], and then all of these features are fused together.

#### 2.5D CNN

**Fig. 2:**
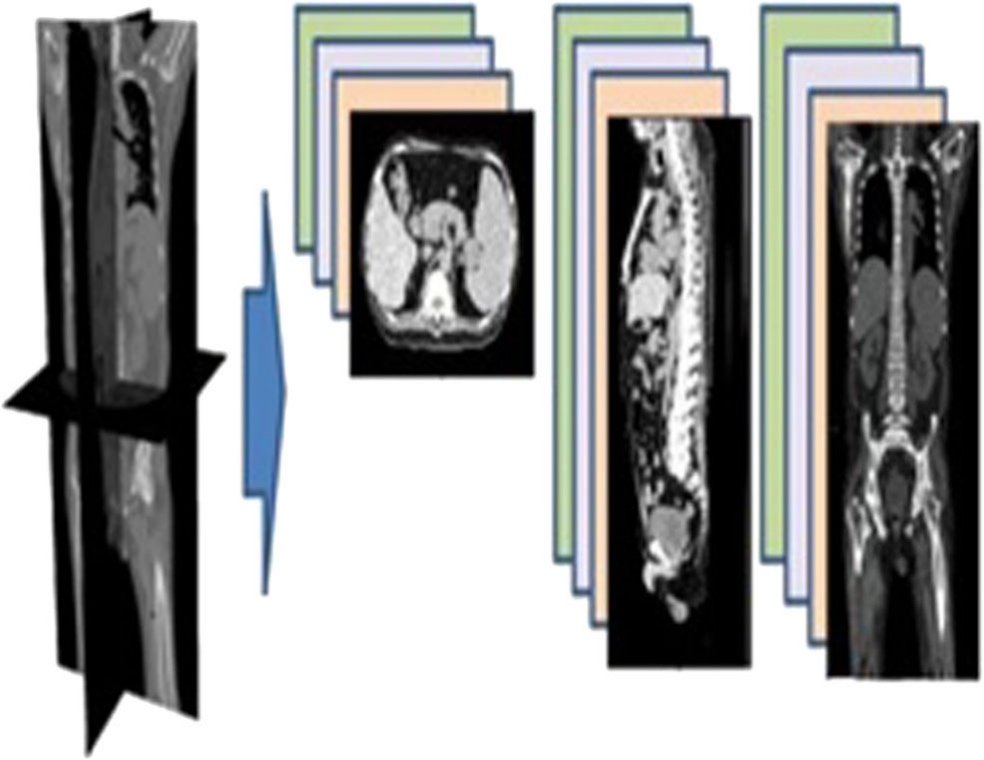
Orthogonal representation of 3D volume [92]

2.5D approaches [54, 60, 65] are inspired by the fact that 2.5D has the richer spatial information of neighboring pixels with less computational costs than 3D. Generally, they involve extracting three orthogonal 2D patches in the *XY*, *YZ*, and *XZ* planes, respectively, as shown in Fig. 2, with the kernels still in 2D.

The authors in [60] applied this idea for knee cartilage segmentation. In this method, three separate CNNs were defined, each being fed with the set of patches, extracted from each orthogonal plane. The relatively low number of training voxels (120,000) and a satisfactory achievement of 0.8249 Dice coefficient proved that a triplaner CNN can provide a balance between performance and computational costs. In [65], three orthogonal views were combined and treated as three channels of an input image.

Moeskops et al. [54] used a 2.5D architecture for multi-task segmentation to evaluate if a single network design is able to perform multi-organ segmentation. They even further expanded the idea by applying different modalities (i.e., brain MRI, breast MRI, and cardiac CTA) for each segmentation task. The choice of a small kernel size of 3 × 3 voxels allowed them to go deeper in the structure and design a 25-layer depth network. This design can be considered as a very deep structure, first proposed by [69]. The final results appear to be in line with previous studies, which demonstrate that a single CNN can be trained to visualize different anatomies with different modalities.

The 2.5D approaches are benefiting from training the system with 2D labeled data, which is more accessible compared to 3D data and has a better match to the current hardware. Moreover, the decomposition of volumetric images into a set of random 2D images helps to alleviate the dimensionality issue [24]. Although the approach seems to be an optimal idea with acceptable performance (slightly better than 2D methods), some people (e.g., [42]) hold the opinion that employing just three orthogonal views out of many possible views of a 3D image is not an optimal use of volumetric medical data. Moreover, performing 2D convolutions with an isotropic kernel on anisotropic 3D images can be problematic, especially for images with substantially lower resolution in depth (the *Z*-axis) [12].

#### 3D CNN

The application of a 2.5D structure was an attempt to corporate richer spatial information. Yet, 2.5D methods are still limited to 2D kernels, so they are not able to apply 3D filters. The use of a 3D CNN is to extract a more powerful volumetric representation across all three axes (*X*, *Y*, and *Z*). The 3D network is trained to predict the label of a central voxel according to the content of surrounding 3D patches. The structure of the network is generally similar to a 2D CNN with the difference of applying 3D modules in each necessary section, for example, in 3D convolutional layers and 3D subsampling layers.

The availability of 3D medical imaging and also the huge improvement in computer hardware has brought the idea of using 3D information for segmentation to fully utilize the advantages of spatial information. Volumetric images can provide comprehensive information in any direction rather than just having one view in the 2D approaches and three orthogonal views in the 2.5D approaches.

One of the first pure 3D models was introduced to segment the brain tumor of arbitrary size [76]. Their idea was followed by Kamnitsas [41] who developed a multi-scale, dual-path 3D CNN, in which there were two parallel pathways with the same size of the receptive field, and the second pathway received the patches from a subsampled representation of the image. This allowed to process greater areas around the voxel, which benefited the entire system with multi-scale context. This modification along with using a smaller kernel size of 3 × 3 has produced better accuracy (an average Dice coefficient of 0.66). On top of that, a lower processing time (3 min for a 3D scan with four modalities) compared to its original design has been achieved.

To address the dimensionality issue and reduce the processing time, Dou et al. in [23] proposed to utilize a set of 3D kernels that shared the weights spatially, which helped to reduce the number of parameters.

To segment an organ from complicated volumetric images, usually we need a deep model to extract highly informative features. But training such deep network is considered as a significant challenge for 3D models. In “Challenges and State-of-the-Art Solutions,” we will address this issue in detail and summarize some of the effective solutions available.

For the subsampling layer, 3D max pooling is introduced which filters the maximum response in a small cubic neighborhood to stabilize the learned features against the local translation in 3D space. This helped to achieve a much faster convergence speed compared to pure 3D CNN thanks to the application of the convolution masks with the same size of the input volume. In [44], Kleesiek et al. performed the challenging task of brain boundary detection using 3D CNN. They applied binary segmentation using a cut-off threshold function and mapped the outputs to the desired labels, and have achieved nearly 6% improvement over other conventional methods. The 3D receptive field of Kleesiek’s model is able to extract more discriminative information compared to 2D and 2.5 since the kernels have learned more precise and more organized oriented patterns as a volume. This is good for segmenting large organs which have more volumetric information than small organs which exist in very few slices of the image.

### Fully Convolutional Network (FCN)

**Fig. 3:**
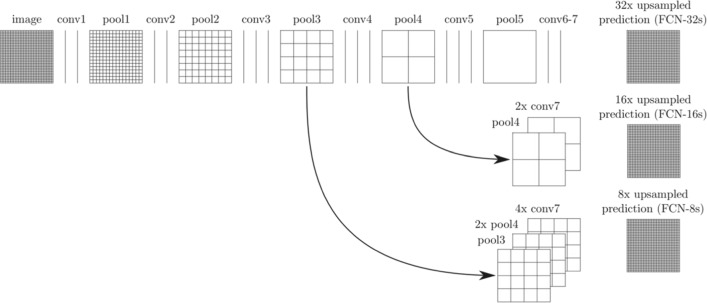
The structure of FCN [50]

In the fully convolutional network (FCN) developed by Long et al. [50], the last fully connected layer was replaced with a fully convolutional layer (see Fig. 3). This major improvement allows the network to have a dense pixel-wise prediction. To achieve better localization performance, high-resolution activation maps are combined with upsampled outputs and passed to the convolution layers to assemble more accurate output.

This improvement enables the FCN to have pixel-wise predictions from the full-sized image instead of a patch-wise prediction and is also able to perform the prediction for the whole image in just one forward pass.

The same experiment as in [89] has been done by Nie et al. but with the application of FCN [57]. As the same modalities and same dataset has been used in both experiments, the result clearly showed the superiority of FCN over CNN by achieving a mean Dice coefficient of 0.885 compared to 0.864.

#### FCN for Multi-Organ Segmentation

Multi-organ segmentation aims to segment more than one organs simultaneously, widely used for abdominal organ segmentation [27]. Zhou et al. [92] used the FCN in a 2.5D approach for the segmentation of 19 organs in 3D CT images. In this study, a pixel-to-label training approach using 2D slices of 3D volume [91] was employed. One separate FCN for each 2D sectional view was designed (totally three FCNs). Ultimately, the segmentation results of each pixel were fused with the results of other FCNs to generate the final segmentation output. The technique produced higher accuracy for big organs such as the liver (a Dice value of 0.937) but yielded lower accuracy while dealing with smaller organs, for instance, the pancreas (a Dice value of 0.553). FCN has also been used for multi-organ segmentation from 3D images [37]. The authors in [66] applied a hierarchical coarse-to-fine strategy that significantly improved the segmentation results of small organs.

#### Cascaded FCN (CFCN)

Christ et al. [15] believed that by cascading the FCNs, the accuracy of liver lesion segmentation could be improved. The core idea of cascade FCN is to stack a series of FCN in the way that each model utilizes the contextual features extracted by the prediction map of the previous model. To do so, a solution is applying a parallel FCN [42, 88] which may increase model complexity and computational cost. The simpler design proposed is to combine FCNs in a cascade manner, where the first FCN segments the image to ROIs for the second FCN, where the lesion segmentation is done. The advantage of using such a design is that separate sets of filters can be applied for each stage and therefore the quality of segmentation can significantly increase. Similarly, in [78], Wu et al. investigated the cascaded FCN to increase the potential of FCN in fetal boundary detection in ultrasound images. The results have shown better performance compared to other boundary refinement techniques for ultrasound fetal segmentation.

In [16], Christ et al. performed liver segmentation by cascading two FCNs, where the first FCN performed the liver segmentation as the ROI for the second FCN which focused on segmenting the liver lesions. This system has achieved 0.823 Dice score for lesion segmentation in CT images and 0.85 in MRI images.

#### Focal FCN

Zhou et al. [93] proposed to apply the focal loss on the FCN to reduce the number of false positives occurred due to the unbalanced ratio of background and foreground pixels in medical images. In this structure, the FCN was used to produce the intermediate segmentation results and then the focal FCN was used to remove the false positives.

#### Multi-Stream FCN

Input images often vary in modality (multi-modality techniques) and resolution (multi-scale techniques). A multi-stream design may allow a system to take benefit from multiple forms of an image from the same organ. In [87], a multi-stream technique was applied to 3D FCN to maximize the utilization of contextual information from various image resolution at the same time applying a multi-modality technique that improved the robustness of the system against the wide variety of organ shape and structure. Unlike [89] which accommodated multiple sources fused the output of each modality at the end of encoder path, here in [87], two down-sampled classifiers were injected to the network to use the contextual information and segment at multiple output layers.

The problem of FCN is that the receptive size is fixed so if the object size changes then FCN struggles to detect them all. One solution is multi-scale networks [42, 77, 83], where input images were resized and fed to the network. Multi-scale techniques can overcome the problem of the fixed receptive size in the FCN. However, sharing the parameters of the same network on a resized image is not a very effective way as the object of different scales requires different parameters to process. As another solution for a fixed-size receptive field, for the images with the size bigger than the field of view, the FCN can be applied in a sliding window manner across the entire image [32].

The FCN which has been trained on the whole 3D images has high class imbalance between the foreground and background, which resulted into inaccurate segmentation of small organs [64, 94]. One possible solution to alleviate this issue is applying two-step segmentation in a hierarchical manner, where the second stage uses the output of the first stage by focusing more on boundary regions [66]. In some of the models multi-stream techniques are used for multi-organ detection (Table 1).

**Table 1:** Comparison of multi-organ segmentation approaches

Approaches	Input dimension	Strategy	Liver	Pancreas
Gibson et al. [27]	2D	−	0.96	0.66
Zhou et al. [91]	2.5D	Orthogonal view of volumetric images	0.937	0.553
Hu et al. [37]	3D	Full 3D	0.96	−
Roth et al. [66]	3D	Hierarchical two-stage FCN	0.954	0.822

### U-Net

#### 2D U-Net

**Fig. 4:**
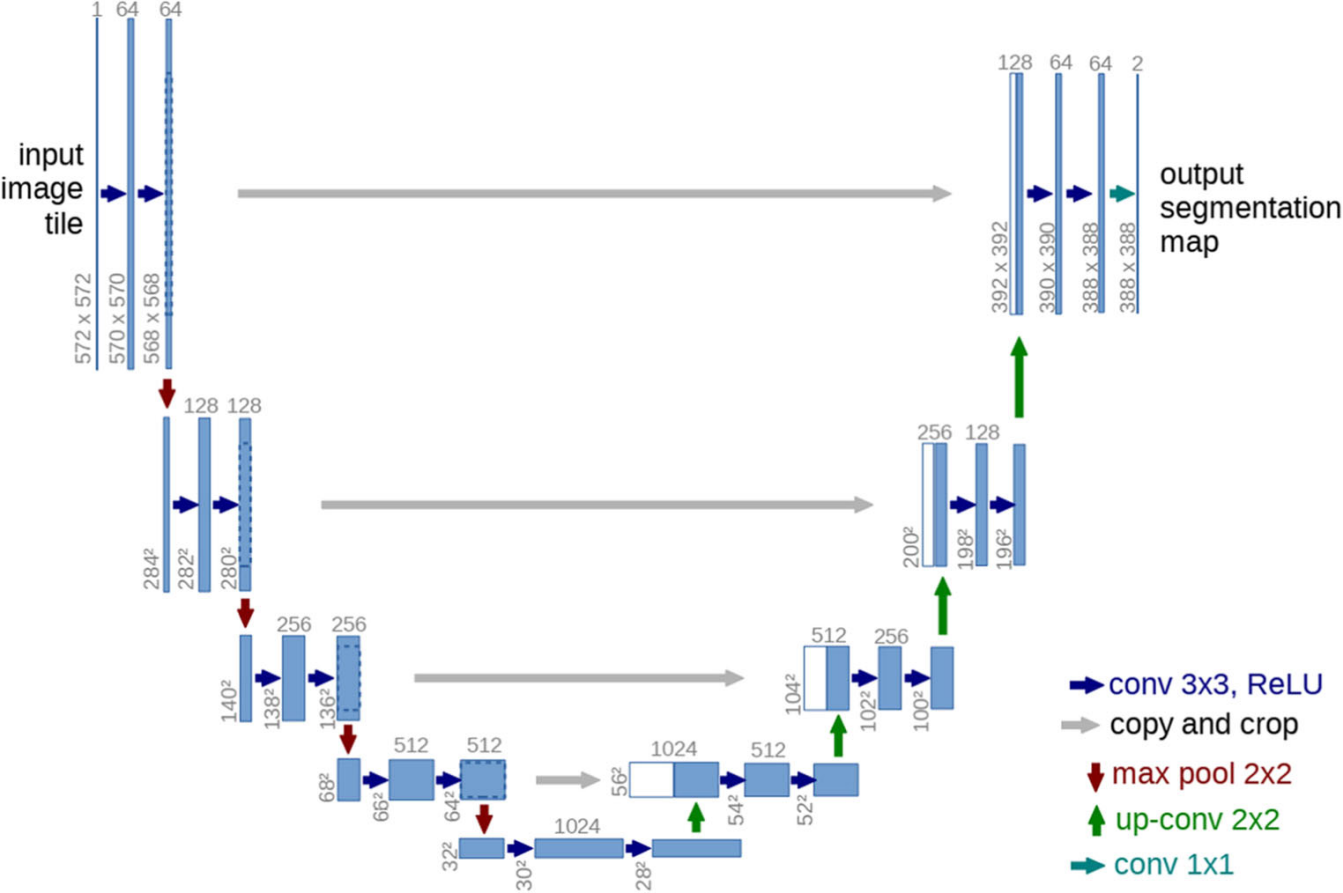
The structure of the U-Net [62]

One of the most well-known structures for medical image segmentation is U-Net, initially proposed by Ronneberger et al. [62] using the concept of deconvolution introduced by [85]. This model is built upon the elegant architecture of FCN. Besides the increased depth of network to 19 layers, U-Net benefits from a superior design of skip connections between different stages of the network [15]. It employs some modifications to overcome the trade-off between localization and the use of context. This trade-off rises since the large-sized patches require more pooling layers and consequently will reduce the localization accuracy. On the other hand, small-sized patches can only observe small context of input. The proposed structure consists of two paths of analysis and synthesis. The analysis path follows the structure of CNN (see Fig. 4). The synthesis path, commonly known as expansion phase, consists of an upsampling layer followed by a deconvolution layer. The most important property of U-Net is the shortcut connections between the layers of equal resolution in analysis path to expansion path. These connections provides essential high-resolution features to the deconvolution layers.

This novel structure has attracted a lot of attention in medical image segmentation and based on which many variations have been developed [17, 31, 86]. For instance, Gordienko et al. [31] explored lung segmentation in X-ray scans with a U-Net structure-based network. The obtained results have demonstrated that U-Net is capable of fast and precise image segmentation. In the same study, the proposed model was tested on single CPU and compared with multiple CPUs and GPUs to evaluate the effect of hardware on model performance. The demonstrated results showed 3 and 9.5 times speedup respectively.

DCAN [11] is another model which applied multi-level contextual information and benefitted from the auxiliary classifier on top of the U-Net. Their design showed 0.8001 of segmentation accuracy on gland segmentation which is almost 2% higher than the original U-Net [62] in a shorter time of 1.5 s per testing image. The improved accuracy is due to the capability DCAN structure to combat the errors of touching object segmentation.

#### 3D U-Net

In an attempt to empower the U-Net structure with richer spatial information, Cicek et al. developed a 3D U-Net model [17]. The suggested model was able to generate dense volumetric segmentation from some 2D annotated slices. The network was able to perform both annotations of new samples from sparse ones and densification of sparse annotated samples. The entire operation of network is redesigned to be able to perform the 3D operation. The average IoU (i.e., Intersection over Union) of 0.863 demonstrated that the network was able to find the whole 3D volume from few annotated slices successfully by using a weighted softmax loss function.

In [44], 3D U-Net was used for vascular boundary detection. The original model of this study was named as HED (Holistic Edge Detection [79]) which was a 2D CNN. Since HED suffered from poor localization power of the small vascular objects, the authors modified the network by adding the expansion path to its structure and successfully overcame this shortcoming. In each stage of the expansion phase, a mixing layer and two convolution layers have been used. The structure of mixing layer is similar to the reduction layer in GooLeNet [73] but with different usage and initialization.

Application of multi-level deep supervision on 3D U-Net-like structures is explored by Zeng et al. in [86]. They divided the expansion part of the network into three levels of low, middle, and up. In the low and middle level, the deconvolution blocks are added to upscale the image to the same resolution of the input. Hence, beside the segmented output of upper level (final layer), the network has two more same resolution segmentation outputs to enhance the final segmentation results.

As one of the shortcomings of 3D U-Net [17], the size of the input image is set to 248 × 244 × 64 and cannot be extended due to memory limitations. Therefore, the ROI-sized input does not have sufficient resolution to represent the anatomical structure in the entire image. This problem can be addressed by dividing the input volume to multiple batches and using them for training and testing [92].

#### V-Net

Probably one of the most famous derivations of U-Nets is the V-Net proposed by Milletari et al. [53]. They applied the convolutions in the contracting path of the network, both for extracting the features and reducing the resolution by selecting appropriate kernel size and stride (kernel size is 2 × 2 × 2, and stride is 2). The convolutions serve as pooling with the advantage of having smaller memory footprint since unlike pooling layers, switches that map the output of pooling layer back to the input do not need to be stored for backpropagation. This is similar to application deconvolution instead of up-pooling [85]. The expansion phase will extract features and expand the concatenated low-resolution feature map and ultimately produce two-channel volumetric segmentation at the last convolutional layer. Then, the output turns to probabilistic segmentation map and passes to voxel-wise softmax for background and foreground segmentation. V-Net has been used in [26] with a larger receptive field (covers 50–100% of the input image) and multi-scale (four different resolutions) and delivered up to 12% higher Dice coefficient compared to original V-Net.

### Convolutional Residual Networks (CRNs)

**Fig. 5:**
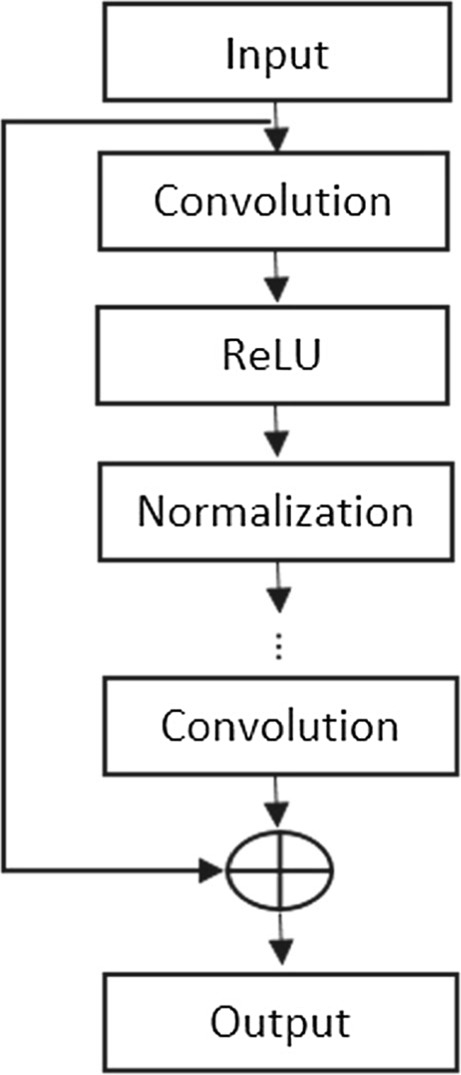
A residual block of CRN. Residual block may have various number and combination of layers inside, depending on the network design

Theoretically, it is proven that deeper networks have higher capability to learn, but deeper networks not only suffer from gradient vanishing problem but also face the more pressing issue of degradation [33]. It means with the depth increasing, the accuracy gets saturated and then rapidly degrades. To take advantage from deeper network structure, He et al. [33] introduced the residual networks which were initially developed for natural image segmentation on 2D images. In this model, instead of consecutively feeding the stacked layers with the feature map, a residual map is fed to every few layers. In other words, the residual maps are skip connections, allowing the network to redirect the derivatives through the network by skipping some layers. This design helped the network to enjoy the accuracy gained from deeper designs (Fig. 5).

Yu et al. further expanded the basic idea of CRN and modified it to a fully convolutional residual network (FCRN) for the accurate task of melanoma recognition and segmentation [83]. The advantage of the proposed FCRN over the original CRN is that it is capable of operating pixel-wise predictions, which is of valuable significance for many segmentation tasks. To get some more benefits over other CNN-based systems, authors perfectly decided to fully utilize both local and global contextual features [43], coming from deeper and higher layers of network respectively. It is addressed by enhancing the model to multi-scale contextual that led to the construction of a very deep FCRN consisting of 50 layers, to segment skin lesions with a Dice coefficient of 0.897 compared to 0.794 for the VGG-16.

Although 2D deep residual networks have demonstrated their capacity in many medical image segmentation tasks [28], as well as general image processing topics [34, 84], yet insufficient studies applied residual learning on volumetric data. Among them, VoxResNet, proposed by Chen et al. [8] is a 3D deep residual network which borrows the spirit of 2D version. They fully benefitted from the main merit of residual networks by designing a 25-layer model to be applied to brain 3D MRI images. The structure consists of VoxRes modules in which the input feature are added to transformed features via a skip connection. Small convolutional kernels are applied since their potential in computational efficiency and representation power already has been proven [69]. To gain a larger receptive area and consequently more contextual information, they employed three convolutional layers with a stride of two that ultimately reduced the resolution of input by eight times. Moreover, they extended the VoxResNet to auto-context VoxResNet which was able to process multi-modality images to provide more robust segmentation. Voxresnet has achieved a segmentation result with Dice coefficients of 0.8696, 0.8061, and 0.8113 for T1, T1-IR, and T2 respectively and 0.885 for the auto-context version. The results demonstrate that increasing the depth not only delivers performance improvement but also provides a practical solution for the degradation problem.

### Recurrent Neural Networks (RNNs)

The RNN is empowered with recurrent connections which enables the network to memorize the patterns form last inputs. The fact that the ROI in medical images usually distributed over multiple adjacent slices (e.g., in CT or MRI), results in having correlations in successive slices. Accordingly, RNNs are able to extract inter-slice contexts from the input slices as a form of sequential data. The RNN structure consists of two major sections of intra-slice information extraction which can be done by any type CNN models, and the RNN, in charge of inter-slice information extraction.

#### LSTM

LSTM [35] is considered as the most famous type of RNN. In a standard LSTM network, the inputs should be vectorized inputs which is a disadvantage for medical image segmentation as the spatial information will be lost. Hence, a good suggestion could be the application of convolutional LSTM (CLSTM) [71, 81] in which vector multiplication has been replaced with the convolutional operation.

#### Contextual LSTM (CLSTM)

In [7], CLSTM applied to the output layer of deep CNN to achieve sharper segmentation by capturing the contextual information across the adjacent slices. Their method achieved significant improvement in DSC of 0.8247 compared to 0.7976 for the famous U-Net structure [62].

Chen et al. in [12] added a bidirectional CLSTM (BDC-LSTM) to a modified U-Net structure CNN. BDC-LSTM is able to capture the sequential data in two directions of *z*^+^ and *z*^−^ rather than single direction. The results have outperformed the pyramid LSTM [72] in which information captured in six directions (*x*^+^,*x*^−^,*y*^+^,*y*^−^,*z*^+^,and*z*^−^), by almost 1%. Although pyramid LSTM moves in six directions, the summation of the six generated outputs from each direction caused spatial information losses. Thus, BDC-LSTM by just moving in *z*-direction perform slightly better.

#### Gated Recurrent Unit (GRU)

GRU is a variation of LSTM in which the memory cells are removed and the structure getting simpler without degradation in performance [13]. Poudel et al. [59] applied a GRU to FCN system and built recurrent FCN (RFCN). Their model was trained end-to-end on segmentation of left ventricular (LV). The RFCN has the advantages of performing both detection and segmentation in a single structure and one pass training for both FCN and GRU.

#### Clockwork RNN (CW-RNN)

The CW-RNN proposed in [45] demonstrated the potential in modeling the long-term dependency with less parameters than a pure RNN. This structure has been applied to muscle perimysium segmentation [80]. Since just a portion of CW-RNN is active at a time, it is more efficient compared to other approaches (100 times less running time than modified CNN [18]), and also the comparison of CW-RNN and U-Net shows a 5% improvement in mean accuracy. It should be noted that the parallelizing the RNN on GPU is a challenging task especially in case of volumetric data [72]. Moreover, having decoupled training for individual modules of RNN has made the training process more complicated and time-consuming. Clearly, RNN approaches have better performance when dealing with bigger organs that have more inter-slice information rather than small lesion segmentation that entire ROI may capture in one slice.

## Network Training Techniques

### Deeply Supervised

The core idea of deep supervision is to provide the direct supervision of the hidden layers and propagate it to lower layers, instead of just doing it at the output layer. This idea has been implemented in [47] for non-medical purposes by adding the companion objective function to hidden layers. Also in GoogLeNet, the supervision was done for two hidden layers of a 22 layers network [73].

Dou et al. in [22] applied deeply supervised approaches to segment the 3D liver CT volumes. This was achieved through upsampling the lower and middle-level features by using deconvolution layers and applying the softmax layer to densify the classification output. Their presented results not only show a better convergence but also lower training and validation error.

In a similar approach [10], three classifiers were injected to classify the mid-level output features from the contracting part of a U-Net-like structure. The classified outputs were used as a regulator at the training phase. The multi-level contextual information in the network helped to improve the localization and discrimination abilities. Moreover, the auxiliary classifiers boosted the back propagation flow of the gradient in the training phase.

### Weakly Supervised

Existing supervised approaches for automated medical image segmentation require the pixel-level (voxel-level in case of 3D) annotation which is not always available in various cases. Also doing such annotation will be very tedious and expensive [39]. In general image processing, this problem eased by using outsource labeling services like Amazon MTurk which obviously cannot be applied to medical images. Alternatively, the use of image-labeled data for instance with a binary label that shows the presence or absence of pattern is a novel approach to address this issue.

This idea was implemented in [2] by employing the “point labels” which are essentially a single pixel location indicating the presence of a nodule to reduce the system dependency to fully annotated images. They took the position of that pixel and extracted the surrounding volume and used it as the positive sample for training by using the statistical information about the nodules. For instance, typically the nodules will be presented in 3–7 consecutive slices and will vary from 3 to 28 pixels in wide. The method achieved a reasonable sensitivity of 80% with weakly labeled samples.

Feng et al. [25] used a CNN for fully automated segmentation of lung nodules in weakly labeled data. Their method is based on the finding of [90] which demonstrated the capability of CNN in identifying discriminative regions. Accordingly, they employed a classification CNN to detect the slices containing nodules, and at the same time, they used the discriminative region features to extract the discriminative regions from the slice, called nodule activation map (NAM). Moreover, a multi-GAP CNN was introduced to take advantages of NAMs from shallower layers with higher spatial resolution same as the idea of [50]. The presented result of 0.55 Dice score was close but less accurate compared to fully supervised approaches. The superiority of deeply supervised methods was expected as they use pixel-level annotation and this provides critical information to deal with various intensity patterns, especially at the edges. However, the proposed method helps to extract the nodule containing areas more automatically compare to [2] which was more established on hard assumptions derived from the statistical information about the nodule size and shape.

### Transfer Learning

Transfer learning is defined as the capability of a system to recognize and employ the knowledge learned in a previous source domain to a novel task [68].

Transfer learning can be done with two approaches, i.e., as fine-tuning the network pre-trained on general images [36] and fine-tuning a network pre-trained on medical images for a different target organ or task. Transfer learning has been proven to have better performance when the tasks of source and target network are more similar, and yet even transferring the weights of far distant tasks has been proven to be better than random initialization [82]. In [78], the weights are taken from a general network (VGG16) and then fine-tuned on prenatal image segmentation in ultrasound. Similarly, in [74], the original weights were taken from a distant application and applied on polyp detection. Therefore, authors had to fine-tune the entire layers. They observed a 25% increment in sensitivity by fine-tuning all layers compared to just the last layer. However, there were some experiments that trained from scratch which also delivered better results compared to fine-tuning a pre-trained network [75].

Transfer learning can be done in three major levels: (1) full network adaption, which is to initialize the weights by a pre-trained network (rather than a random initialization) but update them all during the training [9, 77]. (2) Partial network adaption, which is to initialize the network parameter from a pre-trained network but freeze the weights for first few layers and update the last layers during the training [11, 29, 86]. (3) Zero adaption, which is to initialize the weights for entire network from a pre-trained model and do not change any at all. Generally, zero adaption approach from another medical network is not recommended due to the huge variation in organ’s (target) appearance. It is especially not advised if the sources have been trained on general images. Furthermore, the objects in biomedical images may have very different appearance and size so transfer learning from the models with huge variations in organ appearance may not reduce the segmentation result.

#### Network Structure

However, selection of the approach depends on the network structure as well. For shallower networks, the full adaption yields better performance, yet in deeper structures partially adaptive approaches will reduce the convergence time and computational load [74].

#### Organ and Modality

Another critical element in transfer learning is the target organ and its imaging modality. For instance, in [87], they applied full weight transfer for T1 MRI and partial transfer for T2 modality. The results in [61] show that the fully adaption approach has a better average Dice score (ADS) [38] compared to zero and partial adaption in ultrasound kidney segmentation, since the modality has lots of noises and also organ has huge appearance variation.

#### Dataset Size

The size of target dataset is also a role-playing parameter to decide about the level of transfer learning. If the target dataset is small and the number of parameters is large (deeper networks), full adaption may result in overfitting. Thus, partial adaption is a better choice. On the other hand, if the size of target dataset is relatively bigger, the issue of overfitting will not happen and full adaption can work fine. Tajbakhsh et al. in [74] evaluated the effect of dataset size on a full adaption approach. The results show 10% improvement in sensitivity (from 62 to 72%) by increasing the dataset from a quarter to full size of the training dataset.

## Challenges and State-of-the-Art Solutions

### Limited Annotated Data

Deep learning techniques have greatly improved segmentation accuracy thanks to their capability to handle complex conditions. To gain this capability, the networks typically require a large number of annotated samples to perform the training task. Collecting such huge dataset of annotated cases in medical image processing is often a very tough task and performing the annotation on new images will also be very tedious and expensive. Several approaches have been widely used for addressing this problem. Table 2 summarizes some of the widely used datasets various organ segmentation.

**Table 2:** Summary of widely used datasets for various organ segmentation

Organ	Dataset name	Dataset size	Dimension	Modality	Used in
Abdominal	NIH-CT-82	82 samples	3D	CT	[7, 63, 64]
	UFL-MRI-79	79 samples	−	−	[64]
	Brain MRI C34	−	−	MRI	[54]
Brain	MR Brains	−	−	MRI	[8]
	Find the dataset from Zhang			MRI	[57, 89]
	ADNI	339 samples	3D	PET	[12]
Breast	Breast MRI -34	−	−	T1-MRI	[54]
	INbreast	116 samples	2D	Mammography	[21, 55]
	DDSM-BCRP	158 samples	−	−	[21]
Cardiac	Cardiac CTA	−	−	CT	[54]
Heart	ACDC	150 patients	2D	MRI	[5]
Left ventricular	PRETERM dataset	234 cases	2D	MRI	[48, 59]
Liver	SLiver07	30 samples	3D	CT	[23, 37]
	3DIRCADb	20 samples	3D	CT	[16]
Lung	Lung Nodule Analysis 2016 (LUNA16)	880 patients	2D	CT	[1]
	Kaggles Data Science Bowl (DSB)	1397 patients	2D	CT	[1]
	Japanese Society of Radiological Technology (JSRT)	247 images	2D	CT	[31]
	Lung Image Database Consortium (LIDC)	1024 patients	2D	CT	[3, 14]
Prostate	Promise 2012	−	2D	−	[53]
Skin	ISBI 2016	1250 image	2D	−	[19, 83]
Multiple organ	Computational anatomy	640 samples	3D	CT	[92]

#### Data Augmentation

The most commonly adopted method to increase the size of the training dataset is data augmentation which is the application of a set of affine transformation, e.g., flip, rotate, mirror, to the samples [52] as well as augmenting color (gray) values [30]. In a non-medical experiment, the effectiveness of data augmentation is evaluated and the results show that the traditional augmentation techniques are able to boost the performance up to seven percent [58].

#### Transfer Learning

Transfer learning from the successful models implemented in the same area (or even other areas) is another solution to address this issue. Compared with data augmentation, transfer learning is a more specific solution which depends on many parameters as explained in “Transfer Learning.”

#### Patch-Wise Training

In this strategy, the image is broken down into multiple patches which can be either overlapping or random patches. Random patching may result in higher variance among the patches and better convergence especially in 3D cases where *N* random view of a volume of interest (VOI) is taken as the training sample[65] (if *N* = 3 it is a 2.5D approach) [2]. Yet, random patching has the class imbalance issue and lower accuracy compared to overlapping patches. Hence, it is not advised for small-organ segmentation. Overlapping patches have shown higher accuracy but computationally intensive [23]. The performance relatively depends on the overlapping of the patches and the size of mini-patches [52].

#### Weakly Supervised Learning

As illustrated in “Weakly Supervised,” weakly supervised learning approaches such as [2, 25, 39] are useful to address the issue of insufficient or noisy labeled data. Unsupervised learning methods have also been used to extract more reliable data from a weakly labeled data and then use the extracted annotated data to train the network, which is considered as a hybrid approach for addressing this issue [2].

#### Sparse Annotation

Since fully annotating data is not always possible especially in 3D cases, often we have to use sparsely annotated data. Application of weighted loss functions where the weights for unlabeled data are set to zero is the key to only learn from the labeled pixels in sparsely annotated volume [17].

### Effective Negative Set

Another challenge to overcome is to collect a suitable set of negative samples. To enhance the discrimination power of the network on false positive cases, the negative set must contain cases which are nodule-like but not positive. For instance, the authors in [2] picked random samples from inside the lung area with Hounsfield scale between 400 and 500. This HU range contains the nodule-like samples which are negative. Forty percent of collected samples with this approach are used as positive samples and the rest are used for negative set.

### Class Imbalance

It is very common in medical image processing that the anatomy of interest only occupies a very small portion of the image. Hence, most of the extracted patches belong to the background area, while these small organs (anomalies) are of greater importance. Training a network with such data often leads to the trained network being biased toward the background and got trapped in local minima [51, 53].

A popular solution for this issue is sample re-weighting, where a higher weight is applied to the foreground patches during training [16]. Automatic modification of sample re-weighting has been developed by using Dice loss layer and Dice coefficient [44, 62, 95]. Yet, the effectiveness is limited in dealing with extreme class imbalance [93]. Patch-wise training combined with patch selection can help to address the issue of class imbalance [18]. Fundamentally, during the creation of the training set, a control mechanism can be set to have a balanced number of patches from the background and foreground [52].

Another approach to deal with this issue is sampled loss in which the loss will not be calculated for the entire image and just some random pixels (areas) will be selected for loss calculation [56]. The randomness of candidate selection for loss evaluation is the main drawback of this method which may affect the accuracy of loss calculation.

### Challenges with Training Deep Models

#### Overfitting

Overfitting happens when a model can capture the patterns and regularities in the training set with reasonably higher accuracy compared with unprocessed instances of the problem [30]. Generally, the main reason for overfitting is the small size of the training dataset. Therefore, any solution which can increase the size of data (“Limited Annotated Data”) may help to combat the overfitting problem as well [67].

For instance, creating multiple views of a patch (augmentation) rather than having a single view is proven to have a positive effect in overfitting [25]. Another technique to handle overfitting is applying “dropout” during the training process to discard the output of a random set of the neurons in each iteration from the fully connected layers [70]. Similarly, the drop connect which a newer modification of dropout has been proven to help the overfitting issue [65].

#### Training Time

Reducing the training time and having faster convergence is a core topic of many studies. One of the earlier solutions for this issue is to apply pooling layers which can reduce the dimensionality of the parameters [23]. Recent pooling-based solutions use convolution with stride [69] that has the same effect and but lightens the network. Batch normalization, refers to centering the pixel values around 0 by subtracting them by the mean image[40], is also known as an effective key for faster convergence[5, 17, 43]. Batch normalization is a more preferred approach to improve the network convergence as is not reported to have any negative effects on the performance, while the pooling and down-sampling techniques have led in loosing beneficial information.

#### Gradient Vanishing

Deeper networks are proven to have better performance yet they are struggling with the issue of exploding or completely vanishing of propagated signal (gradient) [42], in other words, the final loss cannot be effectively back propagated to shallow layers. This issue is more severe in the 3D models.

A general solution for gradient vanishing is to have deeply supervised approaches in which the intermediate hidden layers’ output will be up-scaled using deconvolution and passed to a softmax to get the prediction from them. The auxiliary losses together with the original loss of the hidden layer are combined to strengthening the gradient [23, 86, 87].

In approaches with from-scratch-training, careful weight initialization also has improving effect in gradient vanishing as demonstrated in [42], where kernels’ weight were initialized by sampling from the normal distribution.

#### Organ Appearance

The heterogeneous appearance of the target organ is one of the big challenges in medical image segmentation. The target organ or lesion may vary hugely in size, shape, and location from patient to patient [42]. Increasing the depth of network is reported as an effective solution [83].

The ambiguous boundary with a limited contrast between targeting organs and the neighboring tissues is a known inherent imaging challenge. This is usually caused by attenuation coefficient in CT and relaxation time in MRI [23, 46]. Multi-modality-based approaches can address this problem [57, 76, 87, 89]. Moreover, superpixel’s information is known to be helpful for segmenting overlapping or organs at the boundary [2]. Applying weighted loss function with a larger weight allocated to the separating background labels between touching organs is another successful approach for touching objects of the same class [12, 62].

#### 3D Challenges

All the abovementioned challenges in training can be much more severe in dealing with volumetric data due to low-voice variance between the target and neighboring voxels, the larger amount of parameters and also the limited volumetric training data. Having computationally expensive inference is known as an issue discouraging the use of 3D approaches. Applying dense inference proves to significantly decrease the inference time to approximately a minute for a single brain scan [76]. Performing a rule out strategy to eliminate the areas which are unlikely containing the target organ can effectively reduce the search space and lead to faster inference [2].

## Conclusion

In this paper, we first summarized the most popular network structures applied for medical image segmentation and highlighted their advantages over the ancestors. Then, we gave an overview of the main training techniques for medical image segmentation, their advantages, and drawbacks. In the end, we focused on the main challenges related to deep learning-based solution for medical image segmentation. We have addressed the effective solutions for handling various challenges. We believe this article may help researches to choose proper network structure for their problem and also be aware of the possible challenges and the solutions. All signs show that deep learning approaches will play a significant role in medical image segmentation.
